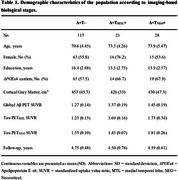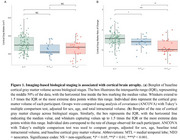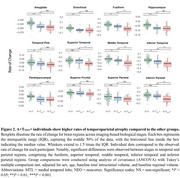# Imaging‐based biological staging associates with brain atrophy in preclinical Alzheimer's disease

**DOI:** 10.1002/alz70856_107663

**Published:** 2026-01-09

**Authors:** Lorenzo Fontura Brasil Barcellos, João Pedro Ferrari‐Souza, Isabela Just de Jesus Vanni, Marco Antônio Albini Valer, Andrei Bieger, Douglas Teixeira Leffa, Firoza Z Lussier, Wagner S. Brum, Cristiano Aguzzoli, Anderson Corin, Marco Antônio De Bastiani, Giovanna Carello‐Collar, Wyllians Vendramini Borelli, Nesrine Rahmouni, Joseph Therriault, Lydia Trudel, Arthur C. Macedo, Pamela C.L. Ferreira, Guilherme Povala, Bruna Bellaver, Diogo O. Souza, Pedro Rosa‐Neto, Tharick A Pascoal, Eduardo R. Zimmer

**Affiliations:** ^1^ Universidade Federal do Rio Grande do Sul, Porto Alegre, Rio Grande do Sul, Brazil; ^2^ University of Pittsburgh, Pittsburgh, PA, USA; ^3^ Universidade Federal do Rio Grande do Sul, Porto Alegre, RS, Brazil; ^4^ Pontifícia Universidade Católica do Rio Grande do Sul, Porto Alegre, Rio Grande do Sul, Brazil; ^5^ Neurology Department, São Lucas Hospital of PUCRS, Porto Alegre, Rio Grande do Sul, Brazil; ^6^ Universidade Federal de Pelotas, Pelotas, Rio Grande do Sul, Brazil; ^7^ McGill University, Montreal, QC, Canada

## Abstract

**Background:**

Alzheimer's disease (AD) diagnostic and staging criteria rely on imaging biomarkers as robust tools for *in vivo* disease assessment. Among these, cortical brain atrophy is recognized as a key hallmark of disease progression. However, rates of cortical atrophy in preclinical stages remain insufficiently explored within current staging frameworks. In a group of amyloid β‐positive (Aβ+) asymptomatic individuals, we investigated the association of the imaging‐based biological AD staging framework with longitudinal brain atrophy patterns.

**Methods:**

We included 162 Aβ+ participants from the A4 Study placebo group with available magnetic resonance imaging (MRI) and positron emission tomography (PET) for amyloid‐β (Aβ) plaques ([^18^F]Florbetapir) and tau ([^18^F]Flortaucipir) at baseline, along with a follow‐up MRI at least 2 years after baseline. Tau positivity in the medial temporal lobe (T_MTL_+) and in the neocortex (T_NEO_+) were determined as tau PET standardized uptake value ratio (SUVR) of 2.5 standard deviations above the mean derived from a separate population (LEARN substudy) of 55 Aβ‐negative individuals. Groups were compared using analysis of covariance (ANCOVA) with Tukey's multiple comparison test, adjusting for relevant covariates.

**Results:**

Demographic characteristics of the population are displayed in Table 1. In cross‐sectional analysis, we observed that baseline cortical gray matter volumes differed significantly only between A+T_NEO_+ and A+T− groups (*p* = 0.042), with no significant differences between other groups (*p* >0.05 for both; Figure 1a). Longitudinally, A+T_NEO_+ individuals exhibited greater rates of cortical atrophy compared to A+T− (*p* <0.001) and A+T_MTL_+ (*p* = 0.007), along with A+T_MTL_+ individuals showing higher rates of cortical atrophy compared to the A+T− group (*p* = 0.042; Figure 1b). Subsequent regional analyses revealed that the A+T_NEO_+ individuals showed higher atrophy compared to both A+T− and A+T_MTL_+ in temporal and parietal regions (Figure 2).

**Conclusions:**

Our findings reveal that the imaging‐based biological AD staging is closely associated with cortical brain atrophy, showing a gradual acceleration in rates of cortical atrophy across stages. Additionally, our results suggest that serial volumetric measurements of temporoparietal brain regions may be sensitive biomarkers of early disease progression. Taken together, our findings provide valuable insights for applying the imaging‐based biological AD staging in preclinical AD.